# Fire up the pyre: inosine thermogenic signaling for obesity therapy

**DOI:** 10.1038/s41392-022-01218-1

**Published:** 2022-11-11

**Authors:** Nienke Willemsen, Stefan Kotschi, Alexander Bartelt

**Affiliations:** 1 Institute for Cardiovascular Prevention (IPEK), Ludwig-Maximilians-University, Munich, Germany; 2 Institute for Diabetes and Cancer (IDC), Helmholtz Center Munich, German Research Center for Environmental Health, Neuherberg, Germany; 3 German Center for Cardiovascular Research, Partner Site Munich Heart Alliance, LMU Hospital, Munich, Germany; 4 Department of Molecular Metabolism & Sabri Ülker Center for Metabolic Research, Harvard T.H. Chan School of Public Health, Boston, MA USA

**Keywords:** Endocrine system and metabolic diseases, Physiology, Target identification

**Fig. 1:**
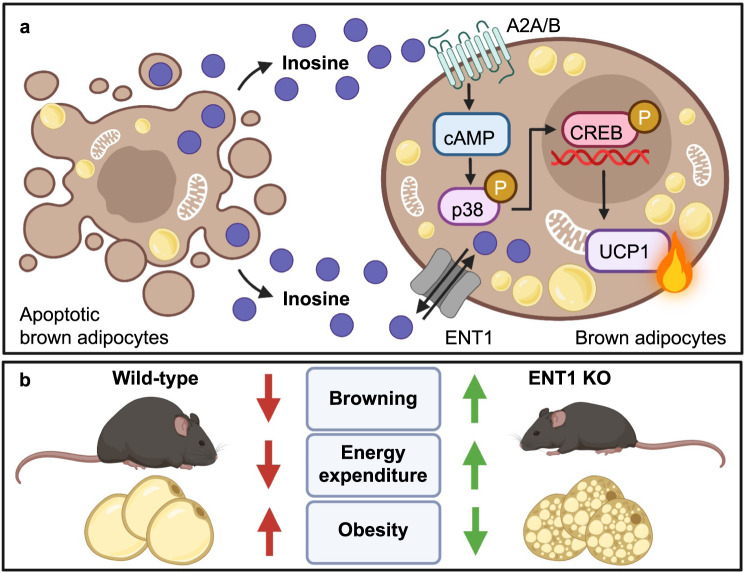
Inosine is a thermogenic stench of death. **a** Inosine released by apoptotic brown adipocytes activates adenosine-2A and -2B receptors (A2A/A2B), stimulating thermogenesis via the cAMP-p38 axis. ENT1 is an inosine transporter that regulates extracellular inosine concentrations. **b** In vivo, ENT1 KO in transgenic mice is associated with higher energy expenditure and browning of adipose tissues as well as protection from obesity. The figure was created with BioRender.com

In a recent study published in *Nature*, Niemann et al. may have discovered a metabolite signaling pathway that could pave the way to new weight loss drugs^1^ (Fig. 1). Obesity and its comorbidities are a major threat to public health, but efficient therapeutics are still scarce.

Brown adipocytes are fat cells that use large amounts of chemical energy in the form of fatty acids and glucose to generate heat, a process called non-shivering thermogenesis.^2^ It is well established that people with higher brown adipose tissue (BAT) activity are generally healthier but how this can be exploited therapeutically is to be determined.^2^ While norepinephrine is the primary stimulator, many other signals have been discovered to participate in the complex physiological regulation of thermogenesis.

Niemann et al. set out to analyze the metabolome released by brown adipocytes undergoing apoptosis, a process linked to adipose tissue remodeling. They identified inosine, a purine-derived nucleoside, as one of the most prominent metabolites.^1^ The supernatant of apoptotic brown adipocytes and specifically inosine by itself, were sufficient to activate thermogenic signaling in brown adipocytes via the GPCR purinergic receptors A2A and A2B.^1^ In mice, injecting inosine increased energy expenditure and induced weight loss,^1^ which is strikingly similar to the natural thermogenic response mediated by norepinephrine. To decipher the mechanism of inosine-mediated thermogenesis, the authors investigated the inosine importer ENT1, encoded by *Slc29a1*. Lack of ENT1 or pharmacological inhibition of ENT1 with dipyridamole replicated the thermogenic effect of inosine.^1^ These mechanisms were also present in human adipocytes, and ENT1 was linked to human obesity on multiple levels.^1^ These results implicate inosine-derived therapeutics or compounds targeting ENT1 as new weight loss drugs.

The work by Niemann et al. supports the concept that the involution of BAT induces cell death, which can be inhibited by adrenergic activation.^3^ It would follow that disuse of BAT in humans during weight gain was linked to higher rates of apoptosis. This would explain the observation that human obesity is associated with diminished BAT activity. While the authors showed an increase in apoptosis markers in BAT from mice exposed to thermoneutrality, the nature and cause of cell death under these conditions remain unclear. Inosine and potentially other signals from dying adipocytes could locally induce thermogenesis and stimulate the formation of more brown adipocytes. It is yet to be established if the same intercellular communication plays a role in the involution of BAT with warmer temperatures and high-fat diets, which is considered to be more similar to the human situation.^3^ Regardless, this work presents an elegant mechanism by which extracellular signals prevent complete loss of BAT during inactivity, thus preserving the plasticity of the tissue in response to cold.

Interestingly, inosine increases intracellular cAMP levels and induces lipolysis in brown adipocytes.^1^ Lipolysis is also implicated in adipose tissue remodeling, as it was previously suggested that lipolysis-associated death of adipocytes leads to the recruitment of macrophages, which would then promote tissue remodeling and browning.^2^ Inosine could be a mediator of the interplay between adipocytes and macrophages, particularly in the context of obesity, in which adipose tissue is inflamed and displays substantial cell death.^2^ Thus, inosine could be driving paracrine communication to ensure tissue remodeling both in pathological (obesity) as well as physiological (cold) adaptations. Of note, extracellular ATP, along with other extracellular nucleotides, is a well-established “stench of decay” signal that is implicated in physiological and immune responses in a wide spectrum of cell types and tissues.

The in vivo effects of inosine treatment on energy metabolism were mimicked by either global genetic deletion or inhibition of ENT1, which both increased extracellular inosine levels. However, ENT1 is rather promiscuous, and other metabolites could be involved in the ENT1 mechanism. The authors showed that either approach increased energy expenditure and Ucp1 levels in adipose tissues, indicating enhanced browning. ENT1 was recently identified as a brown adipocyte marker that is upregulated during cold,^4^ so it is somewhat surprising that ENT1 suppression increases browning. It should be taken into consideration that inosine could also come from and act on non-adipocytes. To address this, the authors analyzed adipocyte-specific ENT1 knockout mice, but obesity in these animals, unlike in the global deletion model, was independent of ENT1. This indicates that the role of ENT1 in obesity is complex. In line with the mouse data, the authors found that in human adipose tissue, ENT1 mRNA levels were inversely correlated with *UCP1* gene expression. Furthermore, they identified the hypofunctional ENT1 missense gene variant p.Ile216Thr that was associated with lower BMI in humans. While these findings support an important role for ENT1 in energy metabolism, more work is needed to fully understand the cell type-specific roles of ENT1 and inosine in human obesity.

The thermogenic effects of inosine place it into the spotlight as a potential weight loss drug. Inosine is already in clinical trials as a neuroprotective agent, as it is metabolized to uric acid, which has anti-oxidative effects. So far, no metabolic benefit has been observed during these oral supplementation studies, but obesity was not specifically investigated.^5^ As the metabolization of inosine prevents its function as an extracellular signal, a more direct application of inosine might be needed to induce its thermogenic effects. Likewise, dipyridamole, which is clinically used as a blood vessel dilator and antiplatelet drug, has not been reported to cause weight loss to our knowledge. It will be challenging to effectively increase extracellular inosine levels in desired tissues in humans to study the weight loss potential of the metabolite.

In conclusion, Niemann et al. have identified inosine as a new metabolically active “death smell” messenger with important implications for our understanding of communication in the tissue microenvironment, adipose remodeling, and energy metabolism.
